# Adoptive immunotherapy using T lymphocytes redirected to glypican-3 for the treatment of lung squamous cell carcinoma

**DOI:** 10.18632/oncotarget.6595

**Published:** 2015-12-14

**Authors:** Kesang Li, Xiaorong Pan, Yanyu Bi, Wen Xu, Cheng Chen, Huiping Gao, Bizhi Shi, Hua Jiang, Shengli Yang, Liyan Jiang, Zonghai Li

**Affiliations:** ^1^ State Key Laboratory of Oncogenes and Related Genes, Shanghai Cancer Institute, Renji Hospital, Shanghai Jiaotong University School of Medicine, Shanghai, China; ^2^ Department of Pulmonary Medicine, Shanghai Chest Hospital, Shanghai Jiao Tong University, Shanghai, China

**Keywords:** lung squamous cell carcinoma, chimeric antigen receptor, glypican-3, CAR T cell

## Abstract

There are unmet medical needs for patients with lung squamous cell carcinoma (LSCC). Therefore, in this study, we explored the antitumor potential of third-generation glypican 3 (GPC3)-redirected chimeric antigen receptor (CAR)-engineered T lymphocytes (CARgpc3 T cells) in tumor models of LSCC. First, we demonstrated by immunohistochemistry (IHC) that GPC3 was expressed in 66.3% of LSCC samples and in 3.3% of lung adenocarcinoma (LAD) samples but not in normal lung tissues. In the presence of GPC3-positive LSCC cells, CARgpc3 T cells were highly activated and increased in number. CARgpc3 T cells could specifically lyse GPC3-positive LSCC cells *in vitro*. In two established LSCC xenograft models, CARgpc3 T cells could almost completely eliminate the growth of GPC3-positive cells. Additionally, the CARgpc3 T cells were able to persist *in vivo* and efficiently infiltrate the cancerous tissues. Taken together, these findings indicate that CARgpc3 T cells might be a novel potential therapeutic agent for the treatment of patients with LSCC.

## INTRODUCTION

Lung adenocarcinoma (LAD, 50–60%), squamous cell carcinoma (LSCC, 30–35%) and large cell carcinoma (5–10%) are the most common histologic subtypes of non-small cell lung cancer (NSCLC) [1, 2]. In the past several years, more and more target therapies have been approved in the treatment of LAD cases [3–6]. On the contrary, no targeted agents had been developed for LSCC before 2015, and chemotherapy therefore continues to be the standard treatment [7, 8]. Fortunately, Nivolumab, a programmed death 1 (PD-1) immune checkpoint inhibitor, was approved this year by the US Food and Drug Administration (FDA) as a second-line treatment for LSCC [9]. However, the overall response rate of patients with LSCC to nivolumab is only approximately 15%. Thus, novel treatments are still urgently needed for patients with LSCC.

Glypican-3 (GPC3) is a member of the glypican family of heparan sulfate (HS) proteoglycans that are attached to the cell surface by a glycosylphosphatidylinositol (GPI) anchor. GPC3 plays an important role in cellular growth, differentiation, and migration [10, 11]. It is generally over-expressed in tumor tissues, including lung cancer, whereas it is almost absent in normal tissues [12, 13]. GPC3-targeted immunotherapeutic strategies that utilize antibodies as well as a peptide vaccine have recently been explored for the treatment of HCC [14, 15].

In recent years, much progress has been made in both basic research and clinical trials regarding cancer immunotherapy with chimeric antigen receptor (CAR)- engineered T cells [16, 17]. CARs could redirect T cells to tumors via extracellular antibody-based domains that are translated in tandem with intracellular T cell signaling moieties in a non-MHC-restricted manner. Recently, we reported that the third-generation GPC3-targeted (GPC3–28BBZ) CAR T cells could eradicate GPC3-positive HCC xenografts in preclinical studies [13]. A clinical study using anti-GPC3 CAR T cells has been registered (NCT02395250) and is ongoing [13]. Given that GPC3 is expressed in LSCC, in this study we explored the potential application of CARgpc3 T cells as a novel intervention for this deadly disease.

## RESULTS

### The expression of GPC3 in LSCC tissues and cell lines

**Figure 1:**
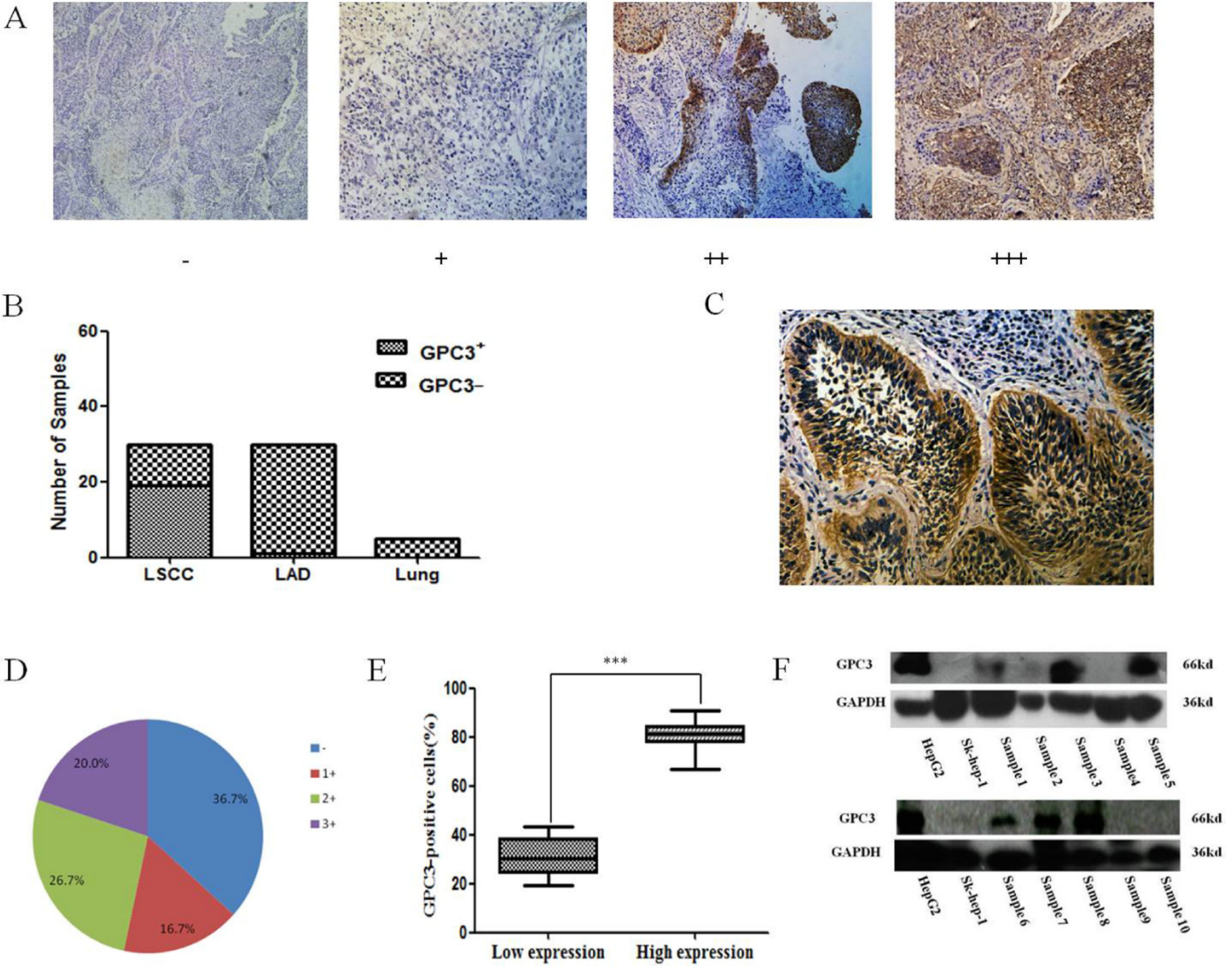
GPC3 expression in lung cancer tissues (**A**) Different expression levels of GPC3 in lung cancer tissues as evaluated by IHC. (**B**) The rate of GPC3-positive staining in LSCC, LAD, and normal lung tissues (Lung). (**C**) The localization of GPC3 expression in lung cancer cells. (**D**) The percentage of GPC3-positive staining in LSCC with different scores is indicated. (**E**) The rate of intratumoral GPC3-positive lung cells in the low GPC3 expression (1+) and in the high GPC3 expression (2+ and 3+) groups (***P < 0.001). (**F**) Expression of GPC3 in human LSCC tissues was detected by western blot. The human HCC cell lines Huh-7 and SK-hep-1 were used as positive and negative controls, respectively.

Because there have been conflicting reports about the expression of GPC3 in LSCC [18–21], we first examined the expression of GPC3 in LSCC, LAD and normal lung tissues by IHC. Similar to previous reports [18, 19], the results of our IHC assay showed that GCP3 was not expressed in normal lung tissue and was expressed in 3.3% (1 out of 30) of LAD cases and 63.3% (19 out of 30) of LSCC cases (Figure 1A–1B). At the same time, we found that GPC3 could be expressed at the plasma membrane in lung cancer cells (Figure 1C). In sections with a high level of GPC3 expression (14 out of 19), GPC3 seemed to be relatively homogeneously expressed in LSCC tissues with a mean rate of GPC3- positive tumor cells equal to 80.79%, which is significantly higher than that in the low-expression group (5 out of 19) in which only 31.18% of cells were GPC3-positive (*P* < 0.001) (Figure 1D–1E).

Thereafter, GPC3 expression in LSCC tissues was further confirmed by western blot. The results shown in Figure 1F demonstrate that the GPC3 protein is expressed in 60% of LSCC tissue samples (6 out of 10; the data for the other five samples are not shown).

Additionally, the expression of GPC3 on the surface of lung squamous cells was determined. Unfortunately, the results of the FACS and western blot analyses confirmed that neither the NCI-H520 cell line nor the SK-MES-1 cell line expressed GPC3 (Supplementary Figure 1A–1B). We found that GPC3 was over-expressed in these two cell lines with transfected GPC3 genes by stable lentiviral transfection methods (Supplementary Figure 1A, 1C). The transfected cell lines were mixed-clone cells (Supplementary Figure 1D–1E).

### Generation of CAR-modified T cells using lentiviral vector transduction

**Figure 2:**
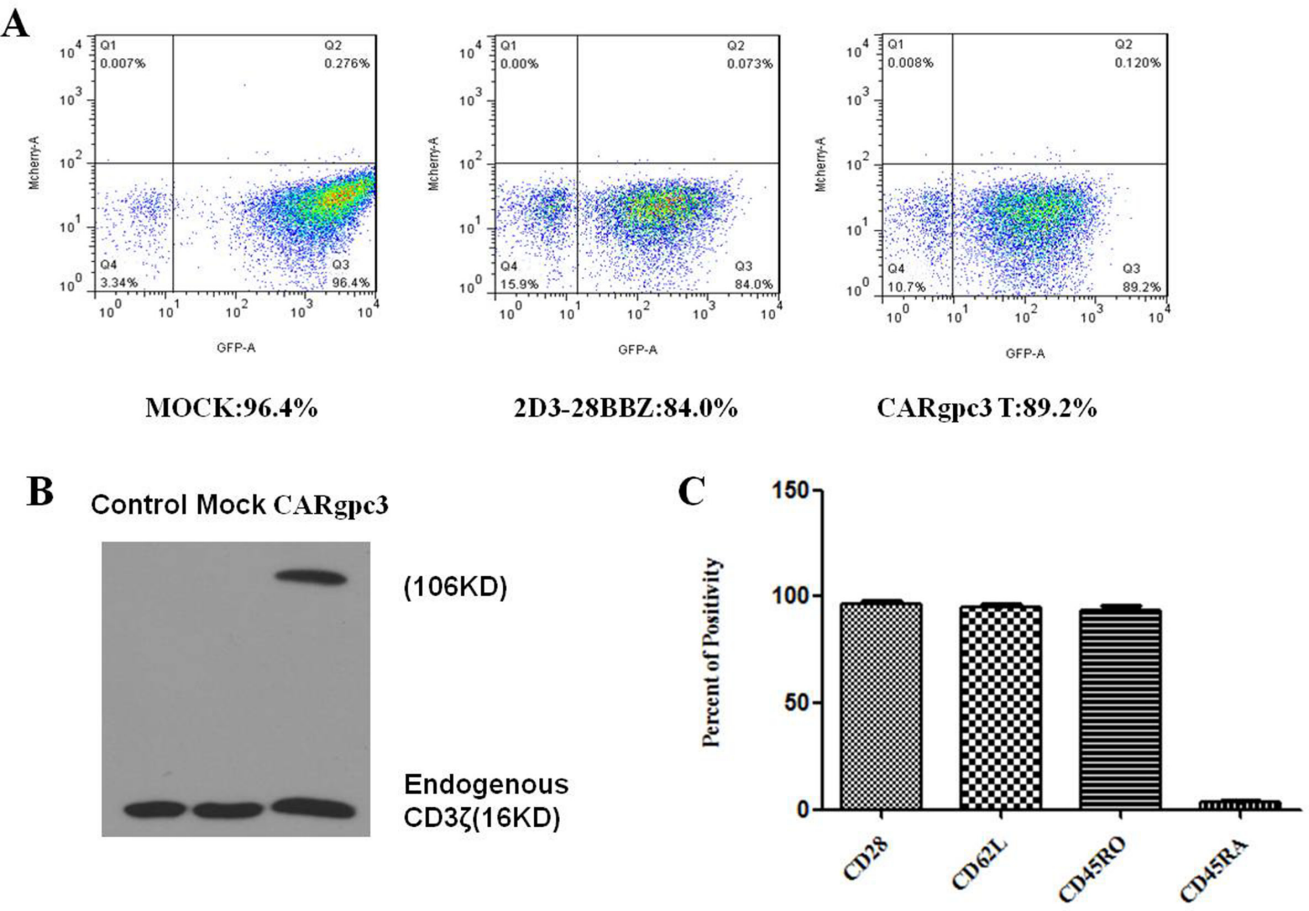
Characterization of CARgpc3 T cells (**A**) The expression of CARs on the surface of T cells was demonstrated through eGFP expression. (**B**) Western blot analysis of CAR expression in T lymphocytes after transduction. Lysates of untransduced T cells (lane 1), eGFP-transduced (lane 2) and CARgpc3 T-transduced (lane 3) T cells were separated by SDS-PAGE. A goat anti-human CD3ζ antibody was used to detect the expression of endogenous and chimeric CD3ζ proteins. (**C**) Flow cytometric analysis of the phenotype of CARgpc3 T-transduced T cells. Fourteen days after the CAR T cells were expanded, the expression of CD28, CD62L, CD45RO, and CD45RA was determined by FACS with the indicated antibodies. The results were concordant in 3 separate experiments.

Primary human CD8^+^ and CD4^+^ T cells mixed at a 1:1 ratio were isolated and transfected with lentiviruses that encode different CARs. According to the FACS analysis, the transduction efficiencies were approximately 85–95% (Figure 2A). The expression of anti-GPC3 CARs was confirmed by western blot. As shown in Figure 2B, in addition to the expression of endogenous CD3 (16 kDa), a CD3 band was observed at the expected molecular mass (106 kDa), which indicates the expression of anti-GPC3 CARs.

To determine whether the CD28/4–1BB-costimulated T cells could reduce apoptosis, we examined Bcl-xL expression in the T cells. We observed that the Bcl-xL protein level was higher in CARgpc3 T cells than in control T cells (including MOCK- or 2D3–28BBZ-transduced T cells) in the presence of LSCC cells with GPC3 over-expression (Supplementary Figure 2). The expansion of CARgpc3 T cells and their increased expression of Bcl-xL in the presence of target cells should be attributed to the activation of the costimulatory signals initiated by CD28 and 4–1BB, both of which have been reported to enhance the activation of human T lymphocytes [22–23].

### Expanded CAR-modified T cells have a central memory phenotype

T cells with a central memory (Tcm) phenotype may be the most appropriate cell type for adoptive cell therapy [24]. Therefore, the phenotype of CARgpc3 T cells was analyzed at 14 days after CAR transduction and culture *in vitro*. The expression of the differentiation markers CD28, CD62L, CD45RO, and CD45RA in CAR T cells was examined by FACS. The results confirmed that the phenotype of CAR T cells was consistent with that of Tcm cells (Figure 2C).

### Cytokine release of CARgpc3 T cells

**Figure 3:**
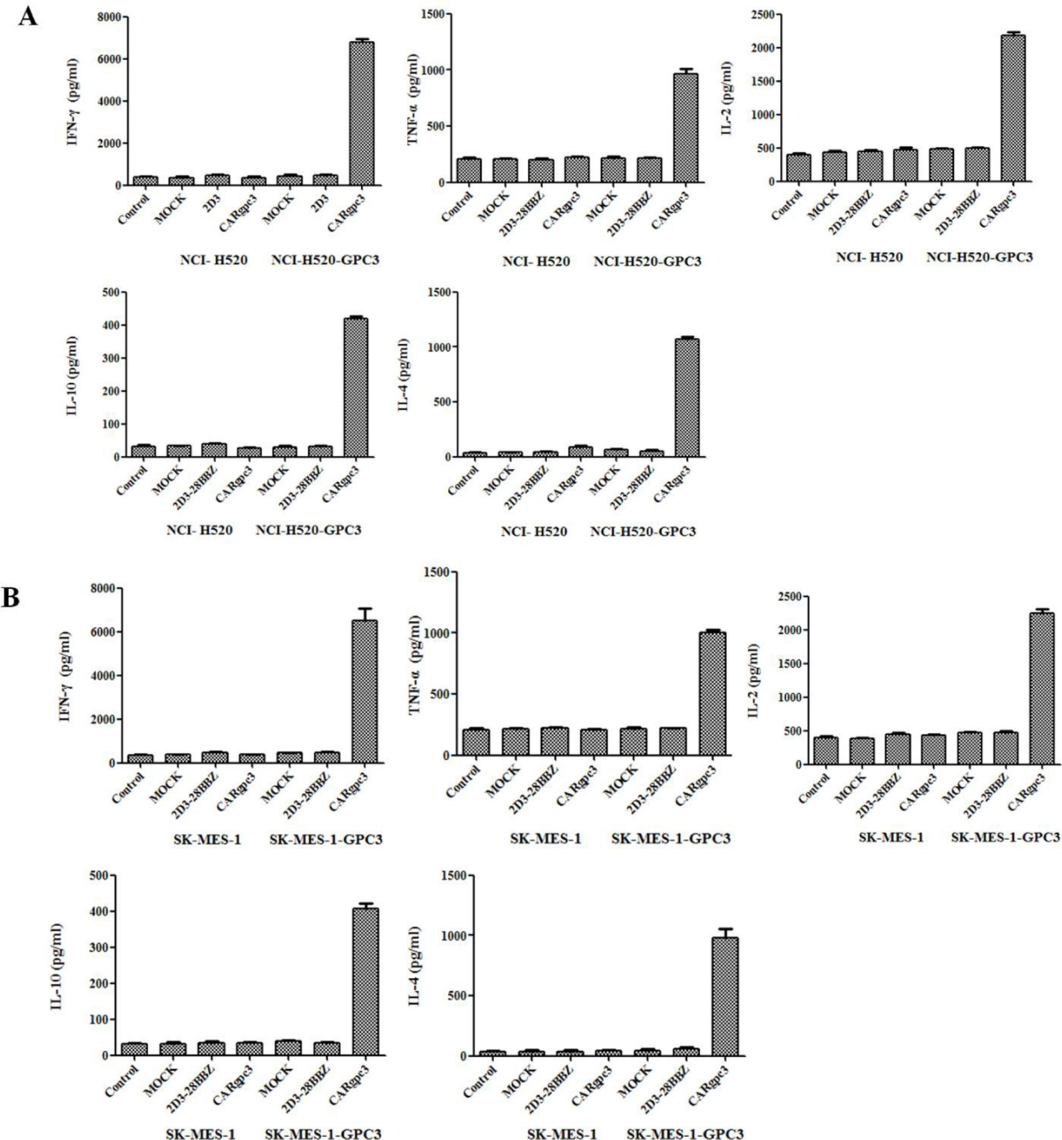
Cytokine release of CARgpc3 T cells In total, 1 × 10^6^ CAR T cells were co-cultured with 1 × 10^6^ tumor cells in 400 μl of medium per well in a 24-well-plate in triplicate. After 24 h, the levels of IFN-γ, IL-2, TNF-α, IL-4 and IL-10 in the supernatants were evaluated by ELISA.

To investigate whether CAR-modified T cells could specifically recognize tumor cells and acquire effector cell functions, a cytokine release assay was performed. As shown in Figure 3A–3B, when they were co-cultured with NCI-H520-GPC3 or SK-MES-1-GPC3 cells but not with NCI-H520 or SK-MES-1 cells, CARgpc3 T cells released a significantly increased amount of IFN-γ, IL-2, TNF-α, IL-4 and IL-10, indicating the T cell activation. By contrast, no significant changes in the secreted cytokines were observed in control T cells (MOCK and 2D3–28BBZ CAR T cells) that were cultured with the target cells.

### Cytotoxicity of CARgpc3 T cells *in vitro*

**Figure 4:**
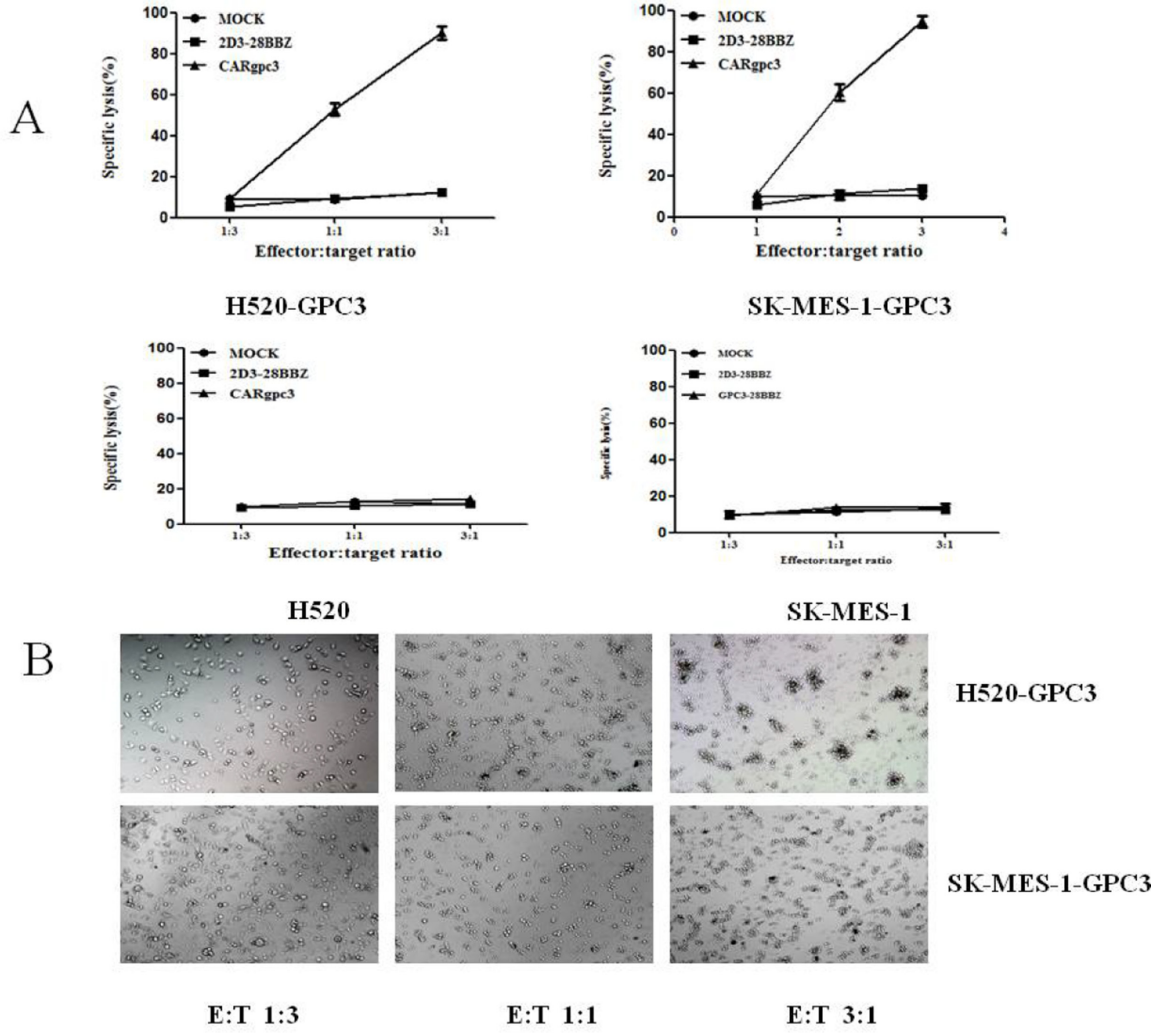
Cytotoxicity of the CARgpc3 T cells against tumor cells with GPC3 over-expression (**A**), (**B**), (C), (D) The cytotoxicity of GPC3-redirected CAR T cells to various lung squamous cell lines at the indicated effector:target (E:T) ratios. (E), (F) Snapshots over time from images of target cells (NCI-H520-GPC3 and SK-MES-1-GPC3 cells) that underwent apoptotic cell death after they were engaged by the CAR T cells.

To determine whether CARgpc3 T cells could specifically recognize and kill GPC3-positive tumor cells, cytotoxicity assays were performed. The results indicated that CARgpc3 T cells could efficiently lyse NCI-H520-GPC3 and SK-MES-1-GPC3 cells but not the parental cell lines (Figure 4A–4D), whereas the control T cells (mock and 2D3–28BBZ) could not initiate the specific lysis of these tumor cells. Additionally, the cytotoxic effect of CARgpc3 T cells on GPC3-positive tumor cells is positively correlated with the effector: target ratios. Concordantly, obvious lysis of the target cells was observed by microscopy when these cells were co-cultured with CARgpc3 T cells (Figure 4E–4F).

The cytotoxicity of CARgpc3 T cells against NCI-H520-GPC3 cells in the effector-to-target ratio of 1:1 was also demonstrated by live-cell time-lapse imaging. Snapshots over time were obtained from live-cell time-lapse images of target cells that underwent apoptotic cell death after they were engaged by the CARgpc3 T cells. From the resultant video, we observed that CAR T cells constantly gathered around the tumor cells until the tumor cells were completely lysed; when this occurred, the CAR T cells moved to another tumor cell until all tumor cells were cleared (Supplementary Video).

### Growth suppression of established GPC3-positive LSCC xenografts by CARgpc3 T cells

**Figure 5:**
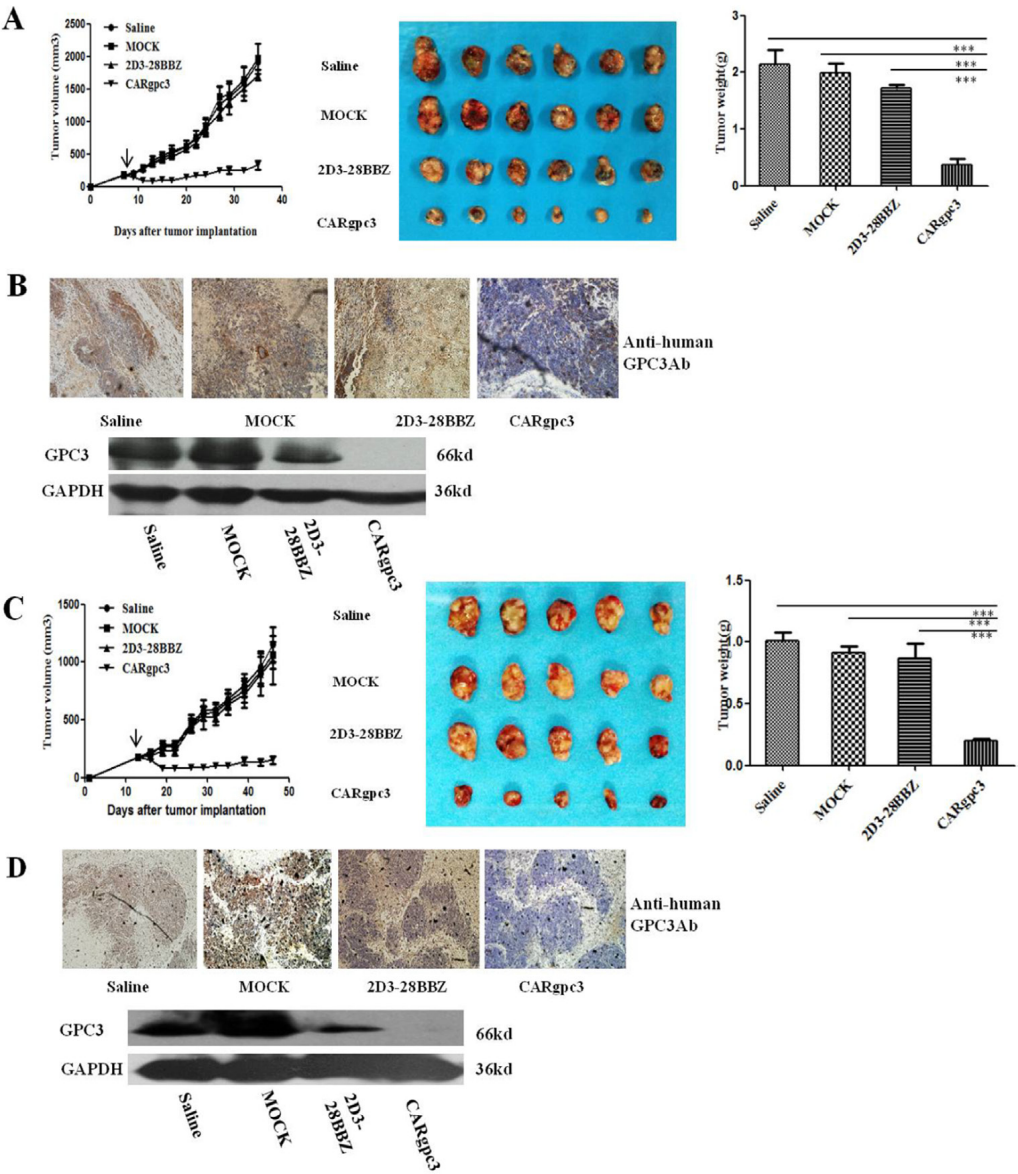
Growth suppression of established s.c. xenografts by CARgpc3 T cells (**A**), (**C**) The tumor volumes and weights were quantified. (**B**), (**D**) CARgpc3 T cells could effectively remove the GPC3-positive LSCC cells. Tumors were collected from mice with NCI-H520-GPC3 or SK-MES-1-GPC3 subcutaneous xenografts treated with CAR T cells or saline. Formalin-fixed, paraffin-embedded tumor sections were consecutively cut and stained for human GPC3 expression (brown) [The images were obtained with a microscope (BX41, Olympus, PA) and camera (DP70) under × 200 magnification. The scale bar is equal to 200 μm]; in addition, a western blot analysis of the expression of GPC3 in NCI-H520-GPC3 and SK-MES-1-GPC3 xenografts is shown.

The antitumor activity of CARgpc3 T cells against established NCI-H520-GPC3- and SK-MES-1-GPC3-based tumor models was then evaluated. Both NCI-H520-GPC3 or SK-MES-1-GPC3 tumors in mice treated with CARgpc3 T cells grew significantly more slowly than those in mice treated with Mock, 2D3–28BBZ CAR T cells or saline alone (*p* < 0.001) (Figure 5A, 5C). The values of the tumor volume were concordant with those of the tumor weights. These results indicated that CARgpc3 T cells efficiently inhibited GPC3-positive LSCC growth *in vivo*.

Next, we examined GPC3-positive tumor cells in the treated tumor sections by IHC and Western blot analysis. The IHC results showed that there were very few GPC3-positive tumor cells in the group of CARgpc3 T cells compared with the control groups (treatment with Mock, 2D3–28BBZ CAR T cells or saline alone). Similar results were observed in western blot assay. Only a very weak band at the expected molecular mass (66 kDa) of GPC3 protein was detected in tumors from of anti-GPC3 CAR-T cells group while strong bind of GPC3 protein was observed in tumors from the other control groups (Figure 7B, 7D). The results of these experiments demonstrated that the CARgpc3 T cells could effectively target and remove GPC3-positive tumor cells *in vivo*.

**Figure 6:**
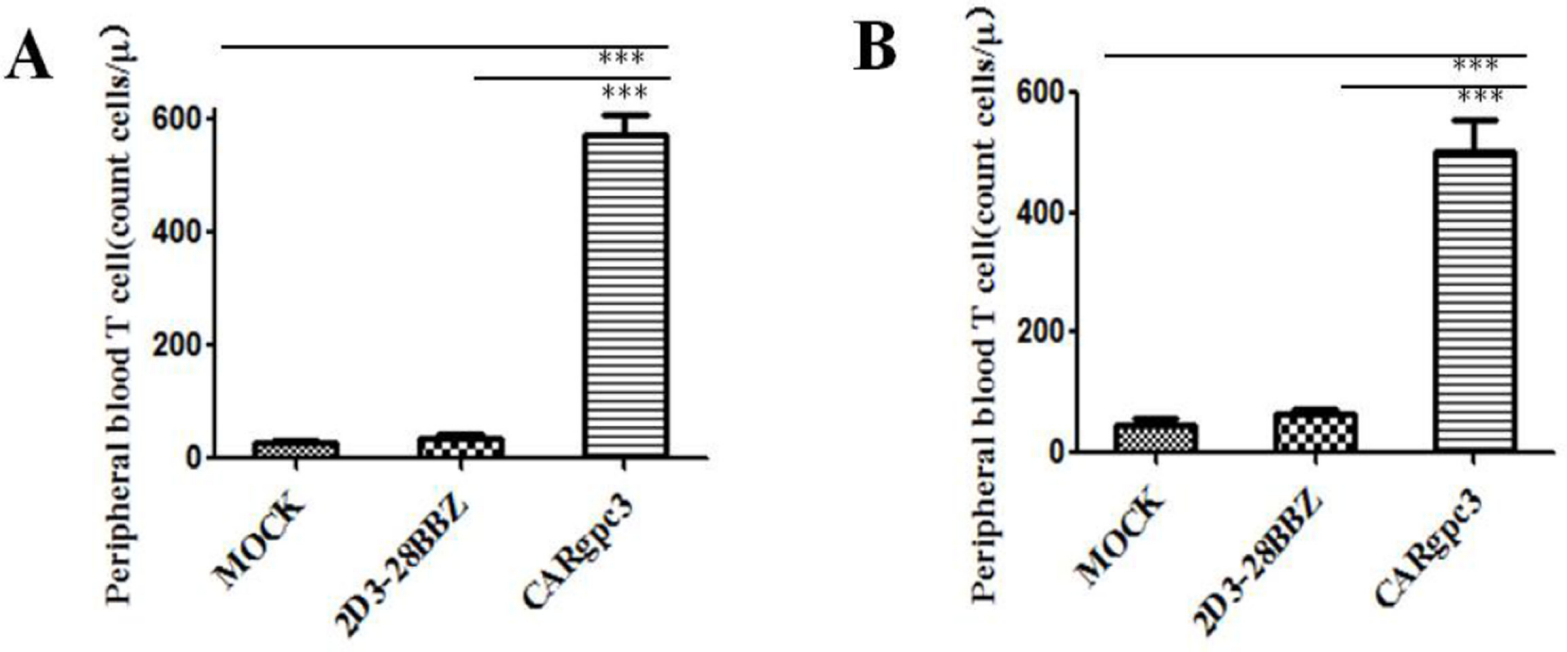
The quantities of GFP-positive CAR T cells in the peripheral blood of mice with s.c. established LSCC xenografts 2 weeks after T cell infusion (**A**) NCI-H520-GPC3 xenografts; (**B**) SK-MES-1-GPC3 xenografts. The mean cell concentration (cells/μL) ± SEM for mice in the treatment groups that received CAR-modified T cells.

**Figure 7:**
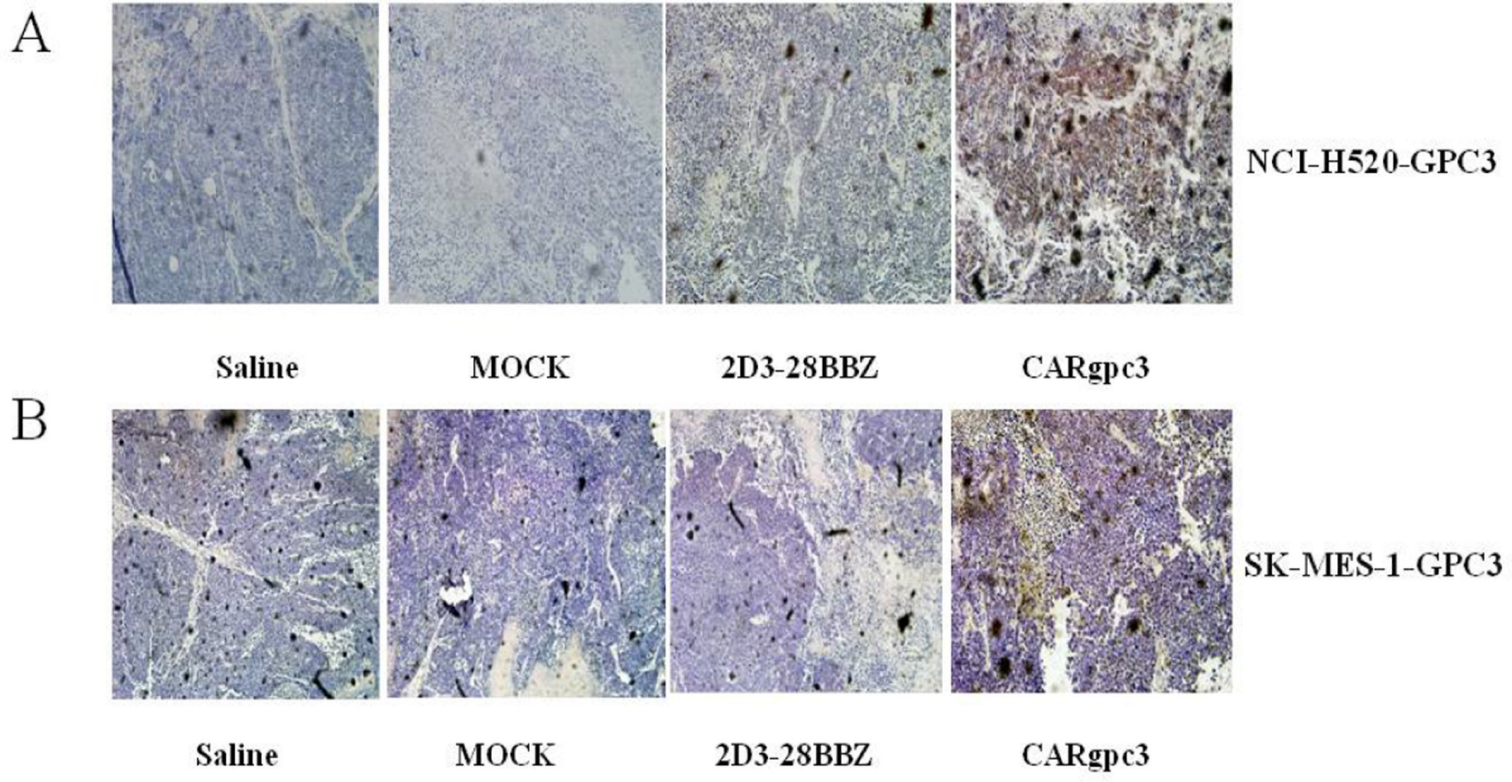
CARgpc3 T cells in GPC3-positive tumors (**A**) NCI-H520-GPC3 xenografts; (**B**) SK-MES-1-GPC3 xenografts. Tumors were collected from mice with subcutaneous xenografts; mice were treated with either CAR T cells or saline. Formalin-fixed, paraffin-embedded tumor sections were consecutively cut and stained for human CD3ζ expression (brown) [The images were obtained with a microscope (BX41, Olympus, PA) and camera (DP70) under × 200 magnification. The scale bar is 200 μm].

### CARgpc3 T cells persist well in the peripheral blood

Previous studies have indicated that the persistence of transferred T cells *in vivo* is highly correlated with tumor regression [25, 26]. Therefore, the number of human T cells in the peripheral blood of mice with s.c. established NCI-H520-GPC3 and SK-MES-1-GPC3 xenografts was quantified two weeks after T cell infusion. The results indicated that the numbers of CAR T cells were significantly higher in mice treated with CARgpc3 T cells compared with mice treated with other T cells (*p* < 0.01) (Figure 6A–6B).

### CARgpc3 T cells can infiltrate GPC3-positive LSCC xenografts

We next investigated whether CAR T cells could infiltrate the tumor site. The results revealed that CARgpc3 T cells did accumulate in residual tumors after intravenous T cell administration (Figure 7A–7B), whereas significantly fewer T cells were detected in the sections of tumors from mice treated with MOCK or 2D3–28BBZ T cells. No specific staining was observed in the tumor sections from mice treated with saline alone.

## DISCUSSION

The recent approvement of the anti-PD1 monoclonal antibody nivolumab for LSCC demonstrates that immunotherapy is a feasible therapeutic approach for patients with LSCC, although only 15% of treated patients benefit from this antibody.

In addition to immune checkpoint inhibitors, T cell-based therapeutics including TCR-T and CAR-T cells are promising interventions for LSCC. Many tumor antigens of interest in lung cancer, including melanoma-associated antigens A3 and A4 and NY-ESO-1, are more frequently expressed in squamous tumors than in non-squamous tumors [27–30]. Furthermore, CD8^+^ effector cells infiltrated LSCC more extensively compared with non-squamous tumors. However, the increased number of tumor-infiltrating CD8^+^ T cells did not correlate with a survival advantage, which suggests an important immune response that may have been due to an immuno-suppressive tumor environment [31–33]. A genomic analysis of squamous cell carcinomas found that many samples exhibited inactivating mutations in the human leukocyte antigen-A class I major histocompatibility gene. This could lead to loss-of-function and reduced expression of tumor antigens, which is a possible immune system-evading strategy [34]. Thus, TCR-T cells that eliminate cancer cells in an MHC-dependent manner do not appear to be very promising for LSCC. Unlike TCR-T cells, CAR-T cells could eliminate cancer cells in an MHC-independent manner. However, CAR-T cells against LSCC have never been reported although CAR T cells that target FAP, EphA2 [35] and EGFR [36] have been developed for the treatment of lung adenocarcinoma. One of the reasons for this might be ascribed to the limited cancer-specific membrane antigens on LSCC.

Previous studies have featured conflicting reports in regard to the expression of GPC3 in LSCC. In this study, we demonstrated that GPC3 is frequently expressed in LSCC but not in normal lung tissues. Several studies have indicated that GPC3 is absent in normal tissues, but one study revealed that a small subset of normal tissues, such as the kidney and gastric glands, expresses GPC3 [12]. Our previous study revealed no obvious GPC3 expression in either the kidney or the gastric glands [13]. Thus, GPC3 should be a rational immunotherapeutic target for LSCC.

The results of this study demonstrated that the third generation of CARgpc3 T cells can be activated and expanded in the presence of GPC3-positive LSCC cells. The *in vitro* cytotoxicity assay further supported that CAR T cells redirected to GPC3 could efficiently eliminate GPC3-positive LSCC cells but not the GPC3-negative LSCC cells. As we know, tumor heterogeneity is one of the major reasons for tumor relapse. The results of the GPC3 expression assay also indicate that GPC3 is not completely homogeneously expressed in LSCC tissues. To elucidate whether the heterogeneous expression of GPC3 might affect antitumor activities, we established LSCC xenografts using the GPC3-transfected LSCC cells in mixed clones and treated the mice with CAR T cells. The results indicate that the CARgpc3 T cells could potently inhibit the *in vivo* growth of the cells of the two GPC3-transfected LSCC xenografts. Additionally, a much higher number of CAR-GPC3 T cells might persist in the peripheral blood and infiltrate the tumor tissues compared with other control T cells, which suggests that GPC3-targeting is important for the activation and expansion of CAR T cells. However, CAR T cells could not completely eradicate the tumor tissues. This result is different from that of our previous study on Huh-7 tumor xenografts [13]. According to IHC and western blot assays, we determined that the residual cancer tissues were almost completely composed of GPC3-negative LSCC cells. Thus, the heterogeneity of the target antigen might be an issue for CAR T cells. Previously, in studies of immunocompetent mice and in human clinical studies, antigen-cross presentation induced by CAR T cells was demonstrated [37–38], which may contribute to the elimination of target-negative cancer cells. Because the mice used in this study were immunodeficient, additional studies should be performed in immunocompetent mice or in clinical trials to elucidate this issue.

Taken together, this study reveals that GPC3 may be a rational target of LSCC and that CARgpc3 T cells might be novel therapeutic agents for patients with LSCC. Therefore, clinical studies using CARgpc3 T cells in patients with LSCC are warranted.

## MATERIALS AND METHODS

### Cell lines and media

Two LSCC cell lines (NCI-H520, SK-MES-1), and 293 T cell lines were obtained from the American Type Culture Collection (ATCC). NCI-H520-GPC3 and SK-MES-1-GPC3 cells were established by lentiviral transduction with the pWPT-GPC3 gene (constructed by our laboratory) into NCI-H520 and SK-MES-1 cell lines, respectively. All cell lines were grown in complete Dulbecco's Modified Eagle's Medium (DMEM) supplemented with 10% FBS (Gibco, UK). Peripheral blood mononuclear cells (PBMCs) derived from human donors were provided by the Shanghai Blood Center. Human primary CD4^+^ and CD8^+^ T cells were cultured in RPMI 1640 medium (Invitrogen, Carlsbad, CA) supplemented with 10% FBS, 100 μg/ml penicillin and 100 U/ml streptomycin. The media were also supplemented with 300 IU/mL recombinant human (rh) interleukin (IL)-2 for 2 days followed by expansion in 300 IU/mL IL-2.

### Immunohistochemistry assay

The use of sections of human lung cancer tissue and normal lung was approved by the Shanghai Chest Hospital and Fudan University Shanghai Cancer Center. The tissue sections contained samples from 60 cases of NSCLC (30 cases of LSCC and 30 cases of LAD) and 5 normal lung tissues from patients who underwent surgical resection at the Shanghai Chest Hospital from 2007 to 2014 and Fudan University Shanghai Cancer Center in 2012 (Clinicopathological characteristics were listed in Supplementary Table 1).

The sections mentioned above were immunostained with an anti-GPC3 antibody at a 1:200 dilution (mAb1G12, BioMosaics Inc.). The levels of GPC3 expression were evaluated using a 4-point scale. A score of - indicates no GPC3 expression, and scores of 1+, 2+, and 3+ signify weak to strong expression of GPC3. The numbers of intratumoral GPC3-positive cells in tissues with low GPC3 expression (1+) and high GPC3 expression (2+ and 3+) were counted. The percentage of GPC3-positive cells in each GPC3-positive section was counted in five different visual fields [13].

Similarly, the expression of GPC3 in the treated tumor tissues was examined with mAb1G12. The infiltration of CAR T cells within tumor tissues was examined using an anti-CD3 antibody at a dilution of 1:50 (Thermo Scientific RM-9107-S0).

The primary antibody was incubated with the tissue sections overnight at 4°C. Secondary goat anti-mouse/rabbit immunoglobulin G (IgG) (ImmPRESS^™^ REAGENT, Vector Laboratories, Inc.) conjugated to horseradish peroxidase (HRP) was then applied. The reactions were developed with DAB (Tiangen Biotech, Beijing, China) chromogen for approximately 3 minutes. The sections were then counterstained with hematoxylin. Appropriate negative controls for the immunostaining assay were prepared in which the primary antibody step was omitted.

### Generation of CAR-expressing T cells

A third generation of a GPC3-specific CAR named CARgpc3, negative control CARs that encodes the truncated CD3ζ (MOCK) or 2D3–28BBZ were constructed as previously described [13]. Recombinant lentiviral particles containing the different CAR genes or a mock control were produced by transfection with polyethylenimine (PEI) [39]. High-titer lentiviral particles were concentrated 30-fold by ultracentrifugation (Beckman Optima^™^ XL-100 K, Beckman, Germany) for 2 h at 28,000 rpm. Negative selection was performed with RosetteSep kits (Stem Cells Technology, Vancouver BC, Canada), which were used for the isolation of primary human T cells (primary human CD4^+^ and CD8^+^ T cells) from healthy human PBMCs. Primary human CD8^+^ and CD4^+^ T cells mixed at a 1:1 ratio were obtained by magnetic-activated cell sorting from healthy human PBMCs. They were subsequently activated by αCD3/αCD28-coated magnetic beads on day 0. On day 1, the T cells were transduced with lentiviruses that encode different CARs.

### Flow cytometric analysis

Flow cytometric analysis was used to detect the expression of GPC3 on tumor cells and to detect the expression of CAR on CD3^+^ T cells. Tumor cells were stained for surface GPC3 expression using a 9C2 mAb (Abcam) and a goat-anti-human IgG-FITC secondary antibody. The transduction efficiency of CAR on CD3^+^ T cells was confirmed by paired GFP-transduced controls. In addition, the central memory phenotype of expanded T cells was examined with a panel of antibodies directed against human CD28, CD45RO, and CD62L (eBioscience). Additionally, a CD3-PerCP/CD4-FITC/CD8-PE kit (BD Bioscience) was used to detect the quantities of circulating human T cells from xenograft-bearing mice treated with CAR T cells.

### *In vitro* cytotoxicity assays

LDH release assays were performed as previously described [36]. Tumor cells were co-cultured with T cells at the different effector:target ratios of 3:1, 1:1 and 1:3 for 16 h. The release of LDH was measured with the CytoTox 96^®^ non-radioactive cytotoxicity kit (Promega, Madison, WI). Live-cell time-lapse imaging was also used to determine the cytotoxicity of CARgpc3 T cells on NCI-H520-GPC3 cells at an effector:target ratio of 1:1.

### Cytokine release assays

The cytokines IFN-γ, IL-2, TNF-α, IL-4 and IL-10, which were secreted by various genetically modified T cells co-cultured with different tumor cells at the effector:target ratio of 1:1 for 24 h, were measured by ELISA (MultiSciences Biotechnology, Hangzhou, China).

### Human LSCC tumor xenograft mouse model

A total of 1 × 10^7^ NCI-H520-GPC3 or SK-MES-1-GPC3 cells were inoculated subcutaneously in the right flank of six- to-eight-week-old NOD/SCID mice on day 0. The mice were administered (intravenously (i.v.) through the tail vein) with 8 × 10^6^ of CAR T cells (transduction efficiencies were approximately 85–95%) at day 7 or 13 when the tumors had grown to approximately 100–200 mm^3^ in volume. At the experimental endpoint, when the tumor volumes reached approximately 2,000 mm^3^ in the control groups, the mice were euthanized. The tumor dimensions were measured with calipers, and tumor volumes were calculated using the formula *V* = ½ (length × width^2^), where the length is the greatest longitudinal diameter and the width is the greatest transverse diameter. NOD/SCID mice were treated and housed according to protocols approved by the Shanghai Medical Experimental Animal Care Commission.

### Western blot analysis

Western blot was performed to verify the expression of CAR proteins. The samples were immuno-blotted with a goat anti-human CD3ζ antibody (Santa Cruz Biotechnology). The blot was then incubated with HRP-conjugated rabbit anti-goat IgG (Sigma). The expression of Bcl-xL in CAR T cells after they were co-cultured with tumor cells for 24 h at an effector: target ratio of 3:1 was examined by western blot using an anti-Bcl-XL antibody (Cell Signaling Technology, USA). β-Actin was used as a loading control.

The expression of human CD3ζ and GPC3 proteins in tumor tissues was examined by western blot using an anti-CD3ζ antibody (Santa Cruz Biotechnology) and an anti-GPC3 antibody (mAb1G12, BioMosaics Inc). Solutions of diluted antibody in PBS-T buffer were supplemented with 5% nonfat dry milk (primary antibody diluted 1:1000; secondary antibody diluted 1:2000). The blots were incubated with horseradish peroxidase-conjugated anti-mouse or anti-rabbit IgG (Kangchen Biotech, Shanghai, China), and the proteins were detected using an ECL western blot analysis system (Pierce, Thermo Scientific, Rockford, IL) in accordance with the manufacturer's instructions.

### Statistical analysis

For statistical analysis, 2-way repeated measures ANOVA with Bonferroni post-tests for tumor burden (tumor volume, tumor weight and photon counts) were used. Differences in the absolute number of various translocated T cells were evaluated by Student's *t*-test. Overall survival statistics were calculated using the log-rank test. GraphPad Prism 5.0 was used for statistical calculations. *P* < 0.05 (*), *P* <0.01 (**) and *P* < 0.001 (***) were considered significant.

## SUPPLEMENTARY TABLE, FIGURES AND VIDEO




