# Supplementary material for: Adoptive immunotherapy using T lymphocytes redirected to glypican-3 for the treatment of lung squamous cell carcinoma
Source: Oncotarget. 2015 Dec 14;7(3):2496–507. doi: 10.18632/oncotarget.6595 (PMC4823050; doi:10.18632/oncotarget.6595)
Supplement: Supplementary file 1 [file oncotarget-07-2496-s001.pdf]

Adoptive immunotherapy using T lymphocytes redirected to glypican-3 for the treatment of lung squamous cell carcinoma

Supplementary Materials

Supplementary Table S1: Clinicopathologic features of 60 patients with lung cancer

Clinicopathologic features	Number of patients
Age (y)	
> 60	41
≤ 60	19
Gender	
M	42
F	18
Pathological type	
Adeno	30
Squamous	30
Tumor histology	
Moderately to poorly	20
Moderately	20
Poorly	16
Poorly to moderately	2
Unidentified	2
Clinical stages	
I	28
II	20
III-IV	12
Lymphatic metastasis	
YES	27
NO	33

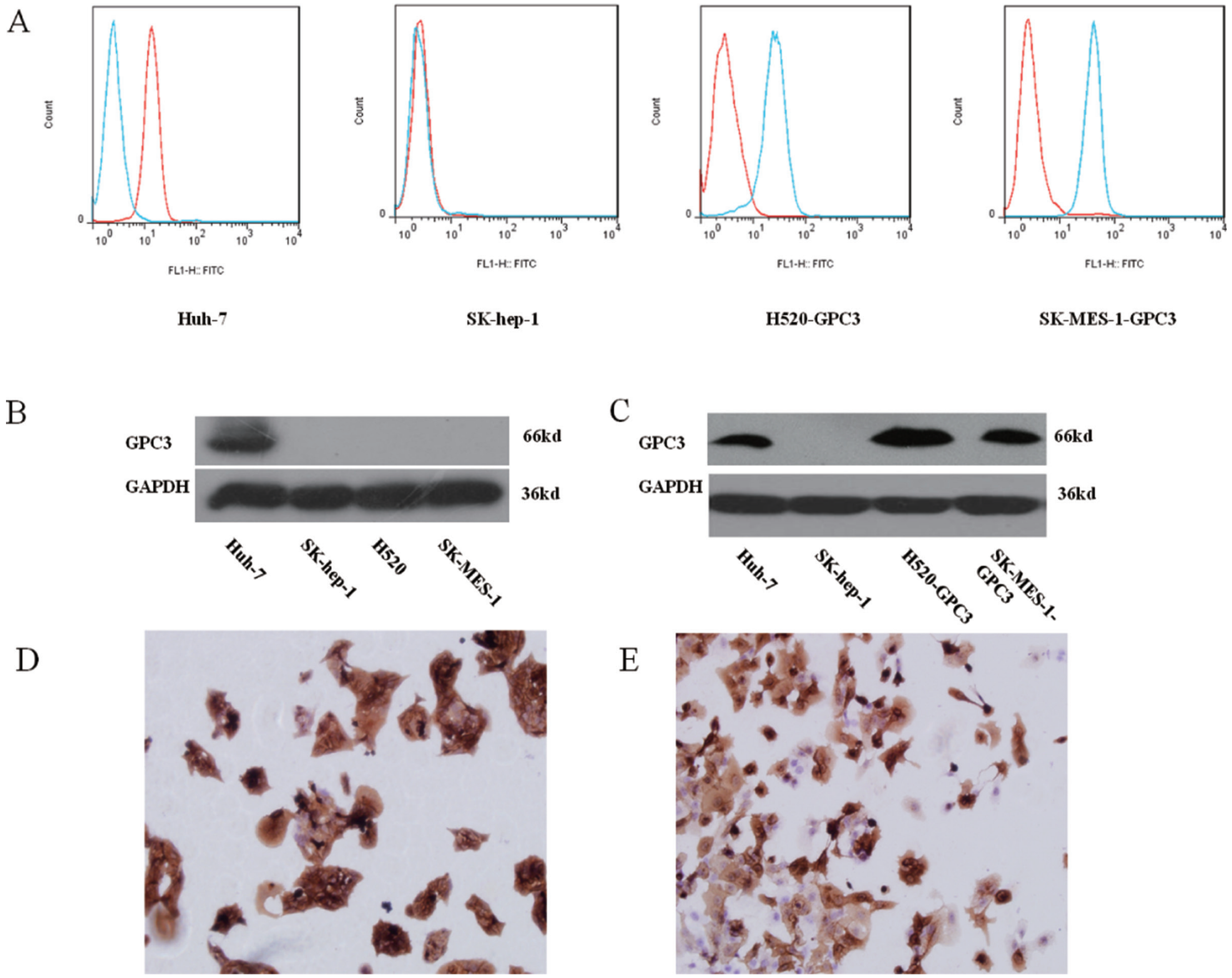

Supplementary Figure S1: GPC3 expression was analyzed in lung squamous cell carcinoma cell lines. (**A**), (**C**) The expression of GPC3 (red line) on the surface of lung squamous cells as detected by flow cytometry. (**B**), (**D**) The expression of GPC3 protein in human lung squamous cell lines was detected by Western blot. The human HCC cell lines Huh-7 and SK-hep-1 served as the positive and negative controls, respectively. (**E**), (**F**) The expression of GPC3 in LSCC cell lines as evaluated by IHC.

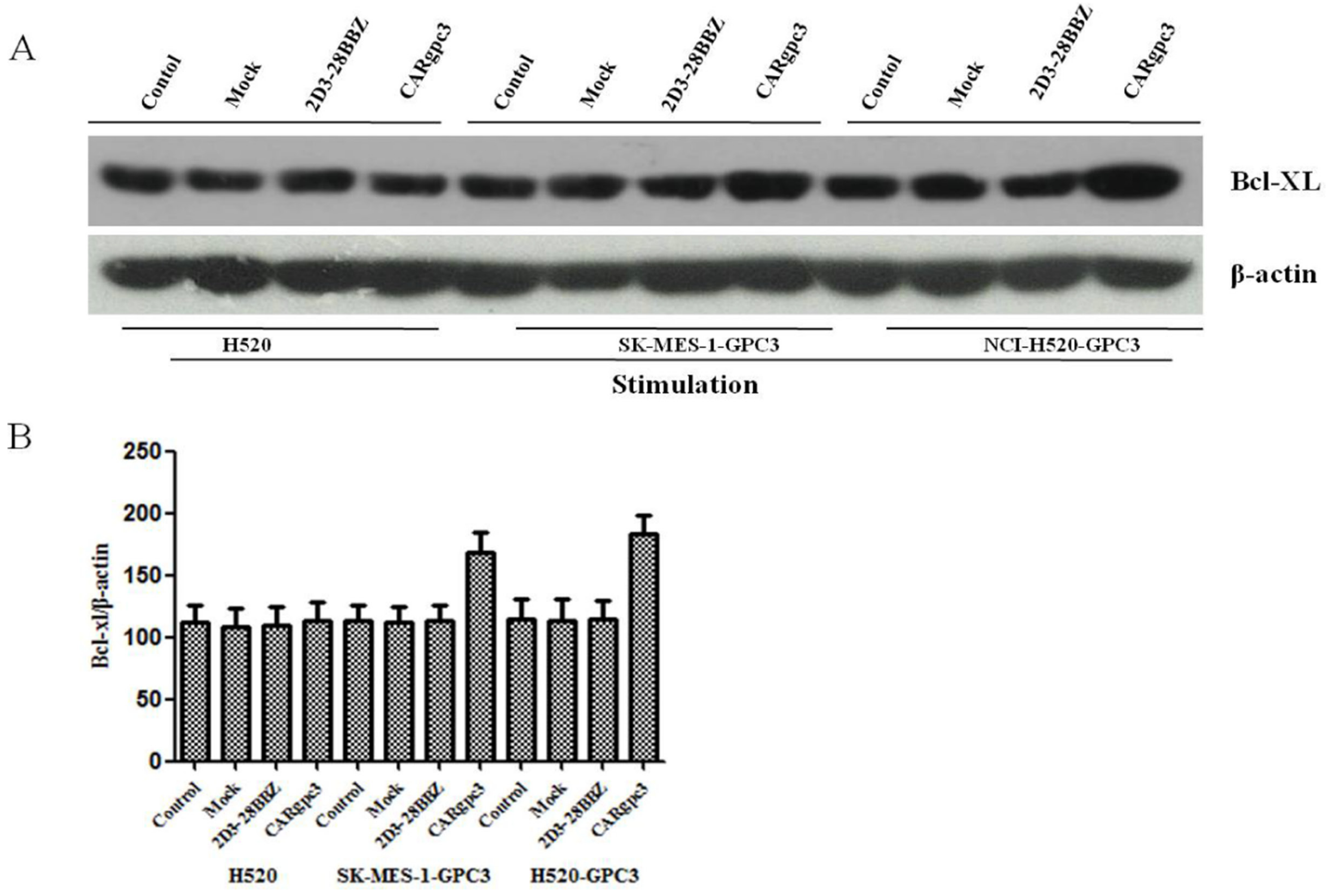

Supplementary Figure S2: Bcl-xL protein in CARgpc3 T cells was up-regulated in GPC3-positive tumor cells. CARgpc3 T cells and the control T cells were each co-cultured with tumor cells (at an effector-to-target ratio of 1:1) for 24 h. The T cells were separated from the tumor cells and subjected to Western blot analysis for the measurement of Bcl-xL protein levels.

Supplementary Video: The cytotoxicity of CARgpc3 T cells against NCI-H520-GPC3 cells in the effector-to-target ratio of 1:1 was also demonstrated by live-cell time-lapse imaging. The incubation time was 24 hours. Snapshots over time were obtained from live-cell time-lapse images of target cells that underwent apoptotic cell death after they were engaged by the CARgpc3 T cells.
